# Tropical lakes as a novel source of oleaginous yeasts with lipid profiles for biodiesel, oleochemical, and nutraceutical applications

**DOI:** 10.1007/s11274-025-04309-7

**Published:** 2025-03-13

**Authors:** Mauricio Ramirez-Castrillon, Tatiana Andrea Benavides-León, Lizeth Vanessa Arcos-Velasco, Kriss Dayana Pantoja-Pulido, Lizbeth Lorena Lopez-Parra, Ana Cristina Bolaños-Rojas, Esteban Osorio-Cadavid

**Affiliations:** 1 Programa de Microbiología, Universidad Santiago de Cali, Calle 5 62-00, Cali, 760035 Valle del Cauca Colombia; 2 Escuela de Microbiología, Facultad de Salud, Universidad Industrial de Santander, Carrera 32 29-31, Bucaramanga, 680002 Santander Colombia; 3 Departamento de Ciencias Químicas y Farmacéuticas, Escuela de Ciencias Aplicadas e Industria Sostenible, Facultad Barberi de Ingeniería, Diseño y Ciencias Aplicadas, Universidad Icesi, Calle 18 122-131, Cali, 760031 Valle del Cauca Colombia; 4 Departamento de Química, Universidad del Valle, Calle 13 100-00, Cali, 760042 Valle del Cauca Colombia; 5 Departamento de Biología, Universidad del Valle, Calle 13 100-00, Cali, 760042 Valle del Cauca Colombia

**Keywords:** Microbial lipids, Poly-unsaturated fatty acids, Petroselinic acid, Single-cell Oil, Lipid-producer, *Aureobasidium* sp, *Rhodotorula* sp, *Clavispora lusitaniae*

## Abstract

Oleaginous yeasts have emerged as promising microbial cell factories for lipid production, offering sustainable alternatives to traditional sources of biodiesel and nutraceuticals. In this study, the lipid accumulation potential of yeast strains isolated from two freshwater aquatic ecosystems in Cali, Colombia, was evaluated to identify novel candidates for biotechnological applications. A total of 56 strains were tested for their oleaginous nature using a gravimetric lipid assay with glucose as a carbon source. Of the assessed strains, 46.15% exceeded 20% lipid yields relative to the dry biomass. Seven strains were selected using glycerol as a carbon source, but only five yeasts were further characterized for their lipid profiles. Molecular identification revealed diverse species, including *Aureobasidium* sp., *Papiliotrema rajashtanensis, Rhodotorula* spp., and *Clavispora lusitaniae*. The selected strains demonstrated unique lipid profiles, with high proportions of monounsaturated and polyunsaturated fatty acids, such as oleic acid (C18:1) and linoleic acid (C18:2). In particular, *Aureobasidium* sp. accumulated uncommon fatty acids such as petroselinic acid under conditions induced by glycerol. This fatty acid, which has a double bond in position 6,7 and a melting point of 33 °C, highlights its potential as an alternative to margarine production, as well as a precursor to sophorolipids, estolide esters, soaps, and plastics. *Rhodotorula* sp. exhibited very long-chain fatty acids such as docosadienoic and docosatrienoic acids in its lipid profile. These findings underscore the biotechnological value of yeasts from lentic aquatic systems as sustainable lipid producers, paving the way for innovations in biofuels, nutraceuticals, and oleochemicals.

**Supplementary Information:**

The online version contains supplementary material available at 10.1007/s11274-025-04309-7.

## Introduction

Fats and oils, or lipids, have been incredibly beneficial to humans for centuries. They have been applied in various industries, including food, cosmetics, medicine, household products, and biofuels (Tao 2007). These oils come from a variety of sources, including plants, animals, or microbes (Tao 2007). Interestingly, the fatty acid composition of these oils varies significantly depending on the source Redondo-Cuevas et al. (2018). Additionally, oil processing and separation methods have been observed to influence its composition (Redondo-Cuevas et al. 2018). A crucial property of fats and oils is oxidative stability, which determines the resistance to spoilage. This stability is influenced by two factors: the degree of saturation of the fatty acids and the location of double bonds within the molecule (Kamal-Eldin 2006). Based on lipid profiles, biotechnological applications of lipids have focused on biodiesel production and the development of Polyunsaturated Fatty Acid (PUFA)-rich microbial cell factories, particularly for omega-3 and omega-6 fatty acids in the nutraceutical industry.

Currently, fish and fish oil serve as the main sources of omega-3 fatty acids, obtained through direct consumption or extraction (Huang et al. 2016; Magoni et al. 2022). However, several drawbacks are associated with fish-derived long-chain PUFAs (LC-PUFAs), including unpleasant odor, presence of saturated fats, contamination with heavy metals, poor oxidative stability, and complex purification processes (Barta et al. 2021; Manikan et al. 2015). Furthermore, increasing demand for LC-PUFA has contributed to overfishing, threatening fish stocks, and leading to unsustainable harvest levels (Magoni et al. 2022). Growing concerns about global warming and the increasing preference for cruelty-free and vegan products have further reduced the appeal of fish oil as a source of PUFAs for dietary supplements, pharmaceuticals and cosmetics (Patel et al. 2020).

Increasing environmental concerns and the depletion of fossil fuel reserves have led to a global shift toward renewable and sustainable alternatives (Alam and Tanveer 2020; Salimon et al. 2012). This trend is evidenced by a decrease in consumer interest in petrochemical products and an increasing preference for products derived from sustainable sources (Alam and Tanveer 2020; Salimon et al. 2012). Biodiesel, derived from oils extracted from edible plants, has been considered a viable alternative fuel. However, the use of food crops for fuel has raised concerns about the future capacity of food supply. Reducing dependence on fossil fuels is crucial, as these resources are both environmentally damaging and finite (Robles-Iglesias et al. 2023; Passoth et al. 2023). As a result, efforts have been directed towards the development of sustainable energy sources and renewable PUFA alternatives for food supplements (Robles-Iglesias et al. 2023).

To address these challenges, single cell oils (SCOs) have emerged as promising sources of sustainable oil for the production of biodiesel and the synthesis of PUFA-rich lipids. Yeast-derived SCOs have garnered significant interest because of their versatile applications as substitutes for both edible and non-edible oleochemical commodities. SCOs are intracellular lipids, primarily composed of triacylglycerols (TAGs) and can be produced by various oleaginous microorganisms, including filamentous fungi, microalgae, bacteria, and yeasts. Oleaginous yeasts are defined as those capable of accumulating at least 20% of their dry cell weight as total lipids, regardless of the environmental conditions that lead to this phenotype (Salvador López et al. 2022). High lipid yield (Y$_{L/X}$) is essential to decrease processing costs per unit of biomass products, while a high growth rate is required to improve overall productivity (Shokravi et al. 2020). Due to their ability to achieve high cell densities rapidly and their diverse metabolic capabilities, oleaginous yeasts are considered ideal candidates for the production of SCO.

Intracellular lipid accumulation is triggered by the depletion of essential nutrients, particularly nitrogen and phosphorus, in the presence of excess carbon (Wierzchowska et al. 2021). Under such conditions, nucleic acid and protein synthesis cellular functions such as these are curtailed, leading to cessation of cell growth. Subsequently, excess carbon is redirected toward lipid biosynthesis through de-novo or ex-novo synthesis pathways (Nunes et al. 2024). These yeasts can utilize different carbon sources, including glucose, xylose, glycerol, starch, cellulose, hemicellulose hydrolysate, and industrial and municipal organic waste, making them attractive candidates for large-scale SCO and biomass production. Furthermore, these yeasts are amenable to genetic manipulation, enabling further optimization. Despite their potential, certain yeast species have limitations in achieving yields close to theoretical lipid yields. These theoretical yields (Y_P/S_) depend on the substrate utilized, with glucose, xylose, and glycerol generally producing approximately 0.32, 0.34, and 0.30 g of lipids/g of substrate, respectively (Caporusso et al. 2021).

The oleaginous phenotype in yeast is strain-dependent (Salvador López et al. 2022). Approximately 160 yeast species (8.2%) (Abeln and Chuck 2021) out of 1,958 known yeast species (Boekhout et al. 2022) have been reported to contain oleaginous yeast strains. Examples of these species include *Cryptococcus* spp., *Yarrowia* spp., *Candida* spp., *Rhodotorula* spp., *Rhodosporidium* spp., *Trichosporon porosum*, and *Lipomyces* spp. Additionally, unconventional yeasts like *Debaryomyces hansenii, Kluyveromyces marxianus, Kazachstania unispora*, and *Zygotorulaspora florentina* also exhibit potential for SCO production (Poontawee et al. 2023). The lipids produced by yeasts consist mainly of monounsaturated and polyunsaturated fatty acids, such as myristic acid (C14:0), palmitic acid (C16:0), stearic acid (C18:0), oleic acid (C18:1), linoleic acid (C18:2), and linolenic acid (C18:3). A major focus of current research is on characterizing the fatty acid profiles of these microbial oils, particularly by improving the production of long-chain PUFAs through metabolic engineering and optimization of the cultivation conditions (Nunes et al. 2024). However, it is fundamental to discover new oleaginous strains and novelty fungal lipids that allow to shorten the path (Hassane et al. 2024). In microalgae, multiple studies have reported oleaginous strains isolated from freshwater environments, with at least 25% of strains demonstrating lipid accumulation up to 40% (g/g dry weight of extracted biomass -DWE-) (Saraf and Dutt 2021; Xu et al. 2020; Wu et al. 2015). Despite their potential, aquatic fungi from freshwater ecosystems remain underexplored in terms of both diversity and biotechnological applications (Grossart et al. 2019; Pagani et al. 2023; Silva-Bedoya et al. 2014). Given these factors, it was hypothesized that focus on the discovery of new oleaginous yeasts from aquatic systems, particularly those with enhanced carbon assimilation capabilities, inhibitor tolerance, and resistance to osmotic stress, could contribute to more efficient and sustainable SCO production processes. Therefore, the objective of this work was to evaluate the lipid-accumulating capacity of wild Colombian yeasts previously isolated from two freshwater aquatic ecosystems.

## Methods

### Chemicals, reagents and culture media

GPY medium was prepared using 10 g/L yeast extract (Scharlau), 10 g/L peptone (M66, Merck), and 20 g/L glucose (Scharlau). GYT medium consisted of 10 g/L yeast extract (Scharlau), 10 g/L tryptone (Sigma-Aldrich), and 20 g/L glucose (Scharlau). Both culture media were used for the reactivation and culture of the strains, in liquid or with microbiological agar (20 g/L, Scharlau) added. The Lipid-inducer medium “B” contained 1 g/L KH$_2$PO$_4$ (Sigma-Aldrich), 0.5 g/L (NH$_4$)$_2$SO$_4$ (Sigma-Aldrich), 0.5 g/L MgCl$_2\cdot$6 H$_2$O (Sigma-Aldrich), plus 50 g/L glucose (Scharlau) or glycerol (Merck). Hexane U.S.P. (Merck), Methanol U.S.P. (Scharlau), sulfuric acid U.S.P. (Merck) were used for the extraction of lipids.

### Yeasts and molecular identification

The yeast strains tested were provided by researchers from the Universidad Santiago de Cali and Universidad del Valle, in Cali, Colombia. These yeasts were isolated from various freshwater systems, including the Cauca and Meléndez rivers, a wastewater treatment plant (PTAR) and a drinking water treatment plant (Puerto Mallarino) (Caicedo-Bejarano et al. 2023). In addition, yeasts were analyzed from two artificial lakes at the Universidad del Valle (Silva-Bedoya et al. 2014). Each strain was reactivated in GPY or GYT media for 3–5 days at 28–30$^\circ$C. All strains were preserved using two methods: storage in sterile mineral oil on a slant for each strain grown with GYT / GYP medium and cryopreservation with 30% (v/v) glycerol dissolved in fresh GYT/GPY medium at -20$^\circ$C.

DNA extraction was performed using the E.Z.N.A. Yeast DNA Kit (Omega, USA) according to the manufacturer’s instructions without modifications. The concentration and absorbance ratio (260 nm / 280 nm) was measured using a NanoDrop 2000 spectrophotometer (ThermoScientific, USA). Yeast identification was carried out by PCR amplification and sequencing of the ITS1-5.8S-ITS2 region and / or D1 / D2 domains of the Large Subunit Ribosomal Gene (LSU). The primers ITS5 (5Â´-GGAAGTAAAAGTCGTAACAAGG-3Â´) and ITS4 (5Â´-TCCTCCGCTTATTGATATGC-3Â´) (Invitrogen, USA) were used for the ITS region, and NL1 (5’-GCATATCAATAAGCGGAGGAAAAG-3’) and NL4 (5’-GGTCCGTGTTTCAAGACGG-3’) for the LSU region. The PCR conditions included, for both regions, an initial step at 95$^\circ$C for 5 min, 30 cycles at 95$^\circ$C for 45 s, 56$^\circ$C for 30 s, and 72$^\circ$C for 1 min, and a final extension step at 72$^\circ$C for 7 min. PCR products were verified using 1.5% (w/v) agarose gel electrophoresis at 100V for 45 min and stained with GelRed (Ref. 41003, Biotium, USA). The gels were visualized under UV light using the Geldoc 2X imaging system (BioRad, USA). PCR products were purified using the ExoSAP-It kit (Thermo Scientific) and sequenced at CorpoGen Corporation (Colombia) following their protocols.

Each sequence was verified by quality and manually edited using BioEdit v7.7 (Mbio, USA). Identification was performed by comparing sequences to type strains in the Genbank and Mycobank databases using pairwise DNA alignment. The identification threshold was set according to Boekhout et al. (2022). If the sequence did not meet the threshold or showed ambiguity, the identification was assigned at the genus level.

### Screening of lipid accumulation and total lipids extraction

The strains were initially grown in GPY or GYT medium for 48 h at 28 °C to obtain metabolically active cells. Subsequently, 10^6^ cells/mL from each strain were transferred to 100 mL of medium B in a 250 mL flask and incubated for 72 h at 28 °C with shaking at 150 rpm (Thermo Scientific). From each culture, six 10 mL aliquots were collected in 50 mL conical tubes. Three aliquots were used for biomass determination and three for total lipid extraction. Each tube was centrifuged at 4500 rpm for 5 min and the supernatant was discarded. The pellet was washed with 1X PBS buffer, followed by a repeat centrifugation step. The pellet was designated as wet biomass.

Lipids were extracted from the biomass using hexane/methanol (2:1, v/v) following the method proposed by Zainuddin et al. (2021). The wet biomass was suspended in hexane / methanol mixture and cell lysis was facilitated by adding 45 mg of 450–600 $\mu$g glass beads (Sigma-Aldrich) and vortexing at maximum intensity for 5 min (Heathrow Scientific). The tubes were then transferred to an ultrasonic bath for 10 min. This procedure was repeated until no visible pellets remained. The tubes were placed horizontally in a a shaker and incubated at room temperature with slow agitation for 25 min. Finally, the tubes were centrifuged at 4500 rpm for 5 min, and the hexane phase was transferred to a pre-weighted glass test tube using an analytical balance (OHAUS 9MV10, México). An additional 5 mL of hexane was added to the original tube and the extraction process was repeated once.

The total lipid performance parameters were determined by evaporating hexane at 37$^\circ$C using a rotary evaporator (Heidolph) and drying at 60$^\circ$C for 24 h. Finally, extracted lipid weight was measured using an analytical balance. Total lipids were expressed as g total lipids by L of culture medium (g/L), lipid content as lipid weight relative to dry weight of extracted biomass (g total lipids / g DWE) and productivity as g lipid/(g DWE $\times$ h). All experiments were carried out conducted in triplicate.

### Gravimetric determination of biomass

For biomass quantification, 10 mL of culture was transferred to a 15 mL conical tube, and centrifuged at 4,233 g for 5 min to remove the supernatant. The pellet was washed twice with 15 mL of 1X PBS buffer. The biomass was dried at 70$^\circ$C until a constant weight was achieved. The dry weight of extracted biomass (DWE) was determined as the difference between the weight of the empty tube and the final measurement. All experiments were performed in triplicate.

### Selection of oleaginous yeasts and Lipid profile

Yeasts were selected for further determination of the lipid profile based on the following criteria: (1) metabolic recovery, discarding strains with low biomass production in GPY, GYT or B media; (2) lipid content of at least 0.2 g / g of DWE, as defined by Angerbauer et al. (2008); (3) lipid concentration of at least 0.5 g/L after 72 h of growth in medium B; (4) molecular identification at the genus or species level; and (5) exclusion of well-known pathogenic species. The selected yeasts were also grown with glycerol as a carbon source at concentrations of 50, 100, or 150 g/L in place of glucose. Five strains that met these criteria were transesterified and analyzed for their lipid profiles.

Before transesterification, experiments were repeated for induction of lipid accumulation and extraction of total lipids. Polytetrafluoroethylene (PTFE) tubes (40 mL) were used instead of plastic tubes to prevent contamination. The lipid extracts were resuspended in 2 mL of hexane after evaporation of the solvent. Transesterification was performed according to Folch et al. (1957), and the final samples were resuspended in HPLC grade hexane (Merck) and transferred to amber HPLC vials for the determination of the lipid profile.

### Gas chromatography-mass spectrometry (GC-MS) analysis and lipid profile

The profiling and quantification of fatty acid methyl ester (FAME) were carried out using a GCMS-QP2020 NX gas chromatograph (Shimadzu), equipped with an HP5-MS column (30 m length, 0.32 mm internal diameter, 0.25 $\mu$m stationary phase diameter). The temperature ramp started at 50$^\circ$C hold for 10 min followed by a 4$^\circ$C/min increase to 150$^\circ$C, then an 8$^\circ$C/min increase to 250$^\circ$C, maintained for 10 min. Helium was used as the carrier gas.

The peak areas of FAMEs were processed using Postrun GC solution software, and the FAME profile was determined by comparing retention times with those of a Supelco C4-C24 FAMEs Mix Standard (Ref. 18919-1AMP, Sigma-Aldrich).

Two chemical parameters were calculated to assess lipid suitability:, Degree of unsaturation (DU) (1) (Anahas and Muralitharan 2015) and Long-Chain Saturated Factor (LCSF) (2) (Anahas et al. 2025). These, and other chemical parameters, were also estimated (see supplementary material) and presented in Table S1, according to the empirical equations proposed by Anahas et al. (2024), and Anahas and Muralitharan (2018).

1 $$\begin{aligned} DU (wt.\%) = (MUFA) + (2xPUFA) \end{aligned}$$

2 $$\begin{aligned} LCSF (wt.\%)= (0.1 X C_{16:0}) + (0.5 X C_{18:0}) + (1 X C_{20:0}) + (1.5 X C_{22:0}) + (2 X C_{24:0}) \end{aligned}$$

## Results

**Fig. 1:**
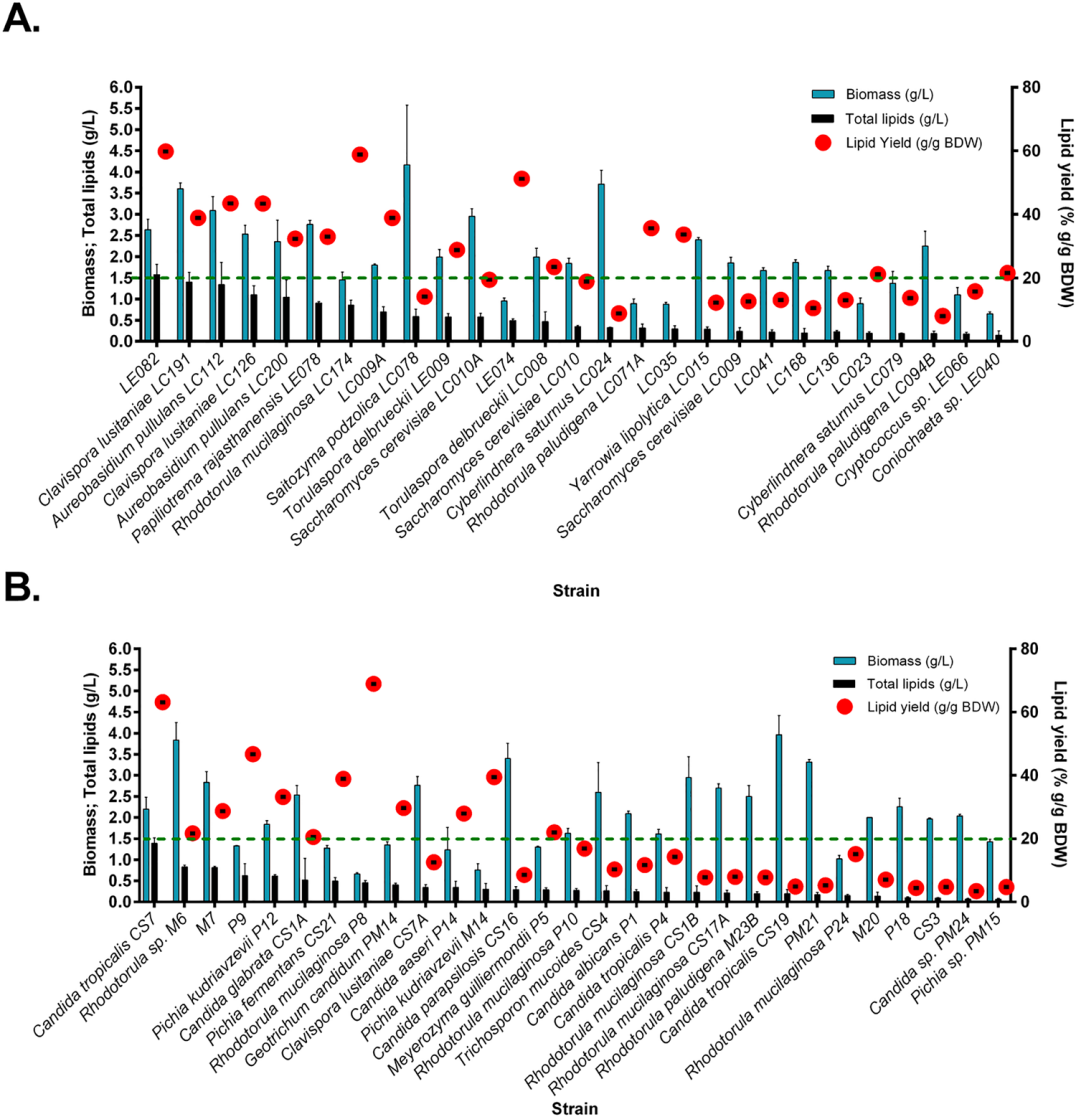
Physiological parameters of evaluated yeast grown on glucose as a carbon source, isolated from artificial lakes (**A**) and other aquatic systems (**B**). The blue bars represent the biomass concentration, the black bars show the total lipid concentration, and the red points represent the lipid yield. All strains with a lipid yield greater than 20% g/g of DWE (represented by a dotted horizontal line) were considered oleaginous. All bars are shown as average ± standard deviation

A total of 56 yeast strains from freshwater environments in Cali, Colombia, were evaluated for lipid accumulation. 27 yeasts were previously isolated from two artificial lakes at the Universidad del Valle (21 in the central lake, 6 in the biological station lake) (Silva-Bedoya et al. 2014), 18 from wastewater (10 from the Navarro South Channel, 9 from PTAR), five from the Melendez River and five from Puerto Mallarino (Caicedo-Bejarano et al. 2023) that were reactivated and grown in culture medium B after 72 h. Figure 1 shows the physiological parameters for all yeasts when induced to accumulate lipids using glucose as the only carbon source. Yeasts were considered oleaginous when the lipid yield was at least 0.2 g total lipids/g DWE, represented by red points above a dotted horizontal line.

The biomass concentration was observed in *Clavispora lusitaniae* LC191 (3.6±0.14 g/L), while the highest lipid concentration was recorded in isolate LE082 (1.58±0.25). Among lentic aquatic environments, such as artificial lakes, 12 strains (46.15%) demonstrated oleaginous characteristics. Notably, *Cl. lusitaniae* LC191, *Aureobasidium* sp. LC112, *Saitozyma podzolica* LC078, and *Cyberlindnera saturnus* LC024 exhibited high biomass production (>3 g/L), qualifying them as strong candidates for SCO production. Strains showing the highest lipid yield included *Cl. lusitaniae* LC126 (43.4% g/g DWE) and *Rhodotorula mucilaginosa* LC174 (58.8% g / g DWE). Although their biomass production was lower than *Aureobasidium* sp. LC112, their high lipid yield made them promising candidates for industrial applications. *Candida tropicalis* CS7 was the only strain from lotic environments that exhibited high lipid accumulation (>60% (g/g DWE).

The growth kinetics for *R. mucilaginosa* LC174, *T. delbrueckii* LE009, *Cl. lusitaniae* LC126 and *Aureobasidium* sp. LC112 were assessed, showing that the stationary phase was reached after 24 h (Fig. S1), except for *Aureobasidium* sp. LC112 showed a diauxic phase, with a change in growing morphology, from yeast-like to pseudohyphae after 72 h. For this strain, the lipid accumulation and melanization increased at least until 120 h (Fig. S2) and their lipid yield against the offered glucose was 0.083 g total lipids/g glucose.

**Fig. 2:**
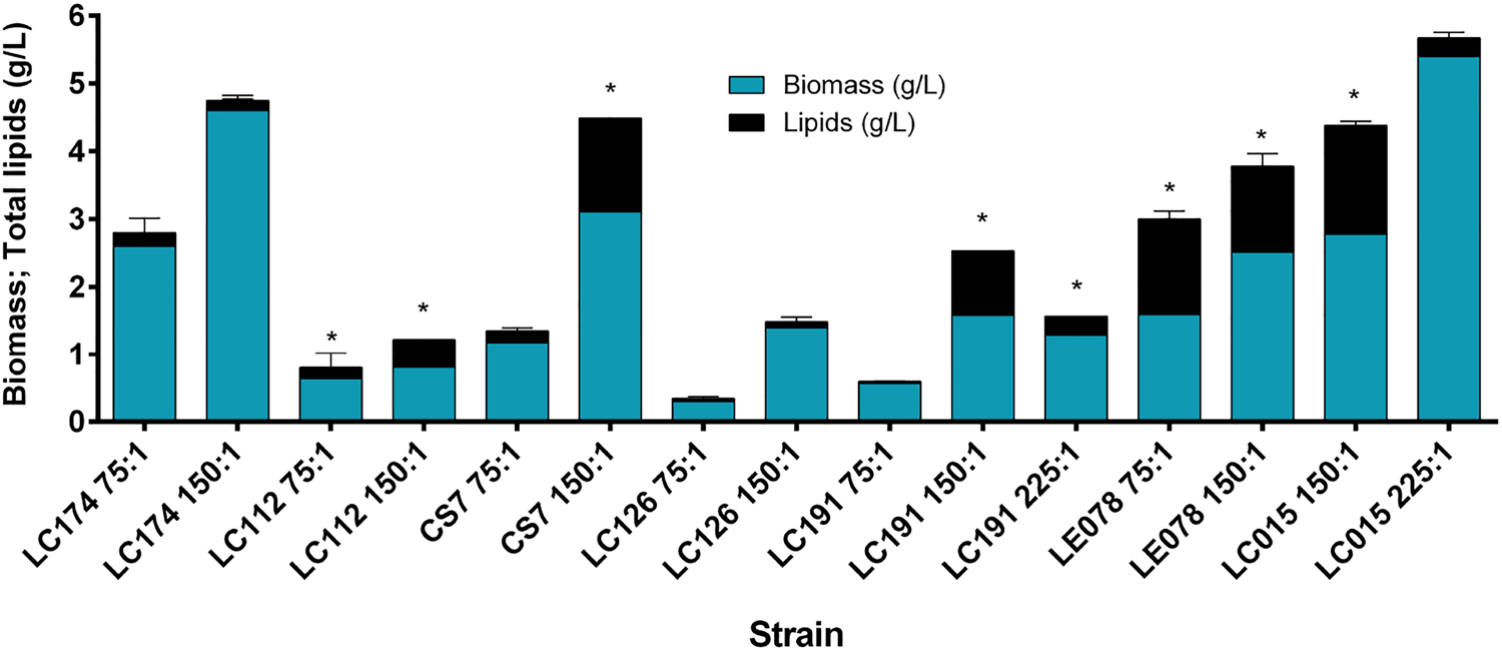
Biomass and total lipids of pre-selected yeasts grown on glycerol as a carbon source. The blue bars represent the biomass concentration, and the stacked black bars represent the total lipid concentration. 75:1, 150:1, and 225:1 represent the C/N ratio. All strains with a lipid yield greater than 20% g/g of DWE were highlighted with an asterisk. All bars are shown as average ± standard deviation

Seven strains were further grown in B-gly medium, with glycerol as the sole carbon source, at C/N ratios of 75:1, 150:1, and 225:1. *Yarrowia lipolytica* LC015 was also included as a reference (Fig. 2). This species is commonly reported in the literature as oleaginous when induced by glycerol (Rywińska et al. 2013; Poli et al. 2014; Kuttiraja et al. 2016). The accumulation varied by strain C/N ratio, with *Aureobasidium* sp. LC112, *C. tropicalis* CS7, *Cl. lusitaniae* LC191, *P. rajashtanensis* LE078 and *Y. lipolytica* LC015 achieving lipid yields greater than 0.2 g/g DWE. *Cl. lusitaniae* LC191 and *Y. lipolytica* LC015 showed lower total lipids using a C / N ratio of 225: 1 compared to 150: 1, suggesting that the concentration of glycerol negatively affects lipid accumulation for these strains.

Table 1 shows the molecular identification of the yeasts evaluated in this study that were not previously reported by Silva-Bedoya et al. (2014); Caicedo-Bejarano et al. (2023), or Villota et al. (2020), or their identification was updated. 20 strains belonged to the Phylum Ascomycota, 12 to Basidiomycota, and 20 were not identified at any taxonomic level. *Aureobasidium* sp. LC112, LC200, *Coniochaeta* sp. LC040, *Papiliotrema* sp. LE066 and *Rhodotorula* sp. M6 showed ambiguity in the identification or did not meet the criteria and were identified only at the taxonomic level of the genus. Unidentified strains were excluded from the next analysis. In total, 11 strains were considered oleaginous; however, within the top six identified yeasts, they showed that they are species related to potentially pathogenic strains, including *C. tropicalis* (Keighley et al. 2024), *C. glabrata* (Katsipoulaki et al. 2024), or *Pichia kudriavzevii* (formerly *C. krusei* (Nguyen et al. 2024). For this reason, these strains were excluded from the next steps of the analysis.

**Table 1:** Yeast identification of preselected strains for lipid accumulation

Strain	Identification	% identity	% coverage	E-value	Region	GenBank Accession
LC008	*Torulaspora delbrueckii*	99.73	98	0.0	ITS	MW130891
LC010	*S. cerevisiae*	100	99	0.0	LSU	JQ672586
LC010A	*S. cerevisiae*	99.83	99	0.0	LSU	PV083825
LC015	*Yarrowia lipolytica*	99.62	98	0.0	LSU	JQ672588
LC024	*Cyberlindnera subsufficiens*	99.66	100	0.0	LSU	JQ672590
LC071A	*Rhodotorula paludigena*	99.23	100	0.0	ITS	MT161373
LC078	*Saitozyma podzolica*	99.18	100	0.0	LSU	PV083826
LC079	*Cyberlindnera subsufficiens*	99.66	100	0.0	LSU	PV083827
LC094B	*Rhodotorula paludigena*	99.22	99	0.0	ITS	MT161374
LC112	*Aureobasidium* sp	98.95	100	0.0	LSU	MN994077
LC112	*Aureobasidium* sp	98.71	100	0.0	ITS	MN994070
LC126	*Clavispora lusitaniae*	99.05	96	0.0	ITS	MW130892
LC191	*Clavispora lusitaniae*	98.75	96	1e-161	ITS	MW130894
LC200	*Aureobasidium* sp	98.71	100	0.0	ITS	MN994071
LE009	*Torulaspora delbrueckii*	99.73	98	0.0	ITS	MW130895
LE040	*Coniochaeta* sp	97.29	100	0.0	LSU	JQ672614
LE059	*Aureobasidium namibiae*	99.81	99	0.0	ITS	MN994074
LE066	*Papiliotrema* sp	98.04	100	0.0	LSU	PV083828
LE078	*Papiliotrema rajasthanensis*	99.83	96	0.0	LSU	JQ672620
M6	*Rhodotorula* sp	99.75	100	0.0	LSU	PV082631

ITS: Internal Transcribed Sequence, ITS1-5.8S-ITS2 region; LSU: Large Subunit; Domain D1/D2 of ribosomal gene 26 S

Table 2 shows the quantified lipid profile of fatty acid methyl esters (FAMEs) for selected yeasts using glucose (50 g/L) and three concentrations of glycerol (100, 50 and 25 g/L). For quantification, the corresponding standard of each FAME present in the SUPELCO mix was compared with the chromatogram and the MS result. In addition, Tables S1 and S2 show the lipid profile in terms of percentage of area relative to total area of all peaks present in each GC chromatogram (see all raw GC chromatograms and quantification processing data in the "Data availability section).

The lipid profile revealed a high percentage of unsaturated fatty acids, with an average of 64.0%, including 36.2% monounsaturated fatty acids (MUFAs). PUFA values ranged from 7.1 to 54.4% (Table S2). In fact, the overall degree of unsaturation (DU) was 91.8%, showing that in most samples the amount of MUFAs and PUFAs is higher than the saturated fatty acids.

The most common FAMEs were palmitic (C16:0), stearic (C18:0), oleic (C18:1$\Delta$ $^9$) and linoleic acids (C18:2$\Delta$ $^{9,12}$), being present in all samples. Furthermore, the concentration of FAMEs decreases 50% on average depending on the initial concentration of glycerol, being notorious in the strain *Aureobasidium* sp. LC112 (for example, palmitoleic acid concentration decreased from 1273 to 672 $\mu$g/mL when the glycerol C/N ratios changed from 225:1 to 150:1, Table 2).

**Table 2:** Fatty acids methyl esters (FAMEs) concentration ($\mu$g/mL) for oleaginous yeasts grown in glucose or glycerol as carbon source

Strain	Carbon source	C/N ratio	C14:0	C16:0	C16:1 $\Delta ^9$	C16:1 $\Delta ^{10}$	C18:0	C18:1 $\Delta ^6$	C18:1 $\Delta ^9$	C18:2 $\Delta ^{9,12}$	C20:0	C22:3 $\Delta ^{8,11,14}$	C22:2 $\Delta ^{13,16}$	C23:0
LC112	Glucose	300:1	451	851	223		300		1381	3258				
LC112	Glycerol	225:1		1375	1273	181	628	194	2563	6657				
LC112	Glycerol	150:1		547	672	57	220	152	1224	3300				
LC200	Glucose	300:1		2114	619		501		3171	13,205				
LC200	Glucose	300:1		1764	548		398		2643	12,512				
LC200	Glucose	300:1		1589	391		381		2377	10,879				
LC126	Glucose	300:1		782	3176		114		2324	2080				
LC126	Glucose	300:1		136			337		38	124				
LC126	Glycerol	225:1	23	961	894	140	437		1903	4904				
LE078	Glycerol	75:1		138			41		52	1023				
M6	Glucose	300:1		1040			1093		594	1676	229	37	149	34
M6	Glucose	300:1		1228			1068		308	2101	318	20	275	61

For *Aureobasidium* sp. LC112, differences in the lipid profile were found, depending on the carbon source. For example, when grown on glycerol, unusual FAMEs were detected, such as methyl (Z)-heptadec-10-enoate (C16:1$\Delta$ $^{10}$) and petroselinic acid (C18:1 $\Delta$ $^6$). Furthermore, the most prevalent FAME that uses glucose was linoleic acid (3258 $\mu$g/mL), representing 50.4% of total quantified FAMEs for *Aureobasidium* sp. LC112. However, the same linoleic acid represented 68% of the total quantified FAMEs for *Aureobasidium* sp. LC200 (12.2 mg/mL). The total PUFAs (including monounsaturated FAMEs) quantified for *Aureobasidium* sp. LC112 were 75% of the total FAMEs, while 85.6% of the PUFAs were quantified for *Aureobasidium* sp. LC200. When changed from glucose to glycerol as a carbon source, *Aureobasidium* sp. LC112 produced 54.3% of linoleic acid (6657 $\mu$g/mL), and 88.7% of total PUFAs.

Regarding *Cl. lusitaniae* LC126, the concentration and number of FAMES were higher in glycerol as a carbon source compared to glucose, with 53% of linoleic acid (4904 $\mu$g/mL), 31.7% of MUFAs and 15% of saturated fatty acid methyl esters (SFA). This lipid profile was completely different when growth with glucose, when the SFA reached more than 50% of the total FAMEs and the DU was only 24%. The LCSF factor was 29.61% for *Cl. lusitaniae* LC126 grown with glucose, which is a promising source of SFA as an energy source, but with a low probability of use as biodiesel feedstock due to their Cold filter plugging point (CFPP) of 76.55$^\circ$C (Table S1).

*Rhodotorula* sp. M6 showed a lipid profile with long-chain FAMEs when growing with glucose as carbon source, including arachidonic acid (C20:0), cis-8,4,11-docosatrienoic acid (DTA, C22:3$\Delta$ $^{8,11,14}$), cis-13,16-docosadienoic acid (DDA, C22:2$\Delta$ $^{13,16}$) and Methyl 20-methyl-heneicosanoate (C23:0). Also, it was detected 13-docosenoic acid (C22:1$\Delta$ $^{13}$) in the lipid profile of M6, a precursor of C22:2$\Delta$ $^{13,16}$, however it was not possible to quantify it. However, the quantity of SFA was almost 50% of the total FAMEs, followed by 25% each of PUFA and MUFA, representing a DU in the range of 62–70%, values of LCSF between 14 and 17%. However, critical parameters for biodiesel suitability, such as CFPP, showed outranged values (28 to 37$^\circ$C) for this strain (see Table S1), compared to Biodiesel standards (Anahas et al. 2024).

## Discussion

The sustainability of the lipid industry is under pressure. Besides the well-established crop-plants based industries for lipid production, the growing demand for oleochemicals and pharmaceutical oils leads to an increase in the extension of crops, competing directly with crops directly related to food (Szczepanska et al. 2022). In this study, novel Colombian yeast strains were evaluated to explore their potential as alternatives to microbial-based manufacturing processes. To our knowledge, this is the first report of oleaginous yeasts in Colombia. Yeasts isolated from aquatic environments, including lentic and lotic systems, were examined, and their ability to accumulate lipids and fatty acid composition profiles was expected to be strain dependent, as proposed by Polburee et al. (2015); Sapsirisuk et al. (2022), with an estimated percentage of oleaginous yeasts ranging between 3–10% (Sitepu et al. 2014, 2019). Previous studies have reported oleaginous yeasts from soils (Alexander et al. 2021; Díaz-Navarrete et al. 2023; Tajdini et al. 2023; Sapsirisuk et al. 2022), mangrove environments (Wongchamrearn et al. 2023; Abdel-Wahab et al. 2023), Lichens (Bai et al. 2023) various plant-derived substrates, such as bagasse (Legodi and Moganedi 2023), flower waste (Kivanc and Otuzbiroglu 2023), fruits (Tatay-Núñez et al. 2024), leaves (Ramírez-Castrillón et al. 2017; Tanimura et al. 2023), and fermented foods or beverages (Poli et al. 2014; Nsa et al. 2020). Regarding freshwater yeasts, only a single report from China has described the detection of oleaginous yeasts in the Yilong lake using primary screening methods (Li et al. 2020).

The results of this study indicate that the rate of oleaginous yeasts differs between the lentic and lotic systems, with an occurrence of approximately 47% in lakes (Fig. 1A). The eutrophication of these lakes, acidic waters, relatively high temperatures (approximately 30 °C), and continuous exposure to birds, lizards, plants, and humans may have contributed to the adaptation of yeast communities to environments with high Chemical and Biological Oxygen Demand. This adaptation may have facilitated the metabolic mechanisms that allow lipid accumulation under these environmental conditions, which are subject to seasonal variations (dry and rainy seasons) (Silva-Bedoya et al. 2014). In contrast, yeasts from lotic systems exhibited a lower capacity for lipid accumulation, independent of species, suggesting that lipid accumulation is influenced by water movement dynamics.

The primary screening methodology used glucose and ammonium sulfate in a C/N ratio of 300:1. A defined medium was selected to establish the C/N ratio stoichiometrically, rather than a complex medium. Glucose was used as the standard carbon source to ensure the reactivation of the majority strains, consistent with the findings of Poontawee et al. (2023), who identified glucose as an efficient carbon source to maximize both biomass and lipid production. Due to the high prevalence of oleaginous yeasts (>20% g/g DWE), selection criteria included not only lipid yield but also biomass concentration and total lipid yield. The selected oleaginous yeasts exhibited total lipid concentrations exceeding 1 g/L and biomass concentrations greater than 2 g/L. Strains were excluded if they could not be definitively identified or classified as pathogenic species. In lotic systems, only *C. tropicalis* CS7 and *Rhodotorula* sp. M6 met these criteria.

The effect of the carbon/nitrogen ratio on lipid accumulation has been extensively documented, with studies indicating that high carbon availability, combined with low nitrogen concentrations, improves lipid accumulation in oleaginous yeasts (Sarantou et al. 2021; Osorio-González et al. 2023; Robles-Iglesias et al. 2023; Lei et al. 2024). However, excessive C/N ratios may adversely affect physiological parameters, including specific growth rates, biomass production, and overall lipid concentration (Abeln and Chuck 2021; Lei et al. 2024). Previous studies showed that C/N 300:1, as applied in this study, effectively induces lipid accumulation and facilitates selecting promising yeast strains (Huang et al. 2016; Ramírez-Castrillón et al. 2017; Lei et al. 2024). More experiments should be conducted to improve the performance of pre-selected strains. For example, *Aureobasidium* sp. LC112 exhibited a lipid yield ($Y_{p/s}$) of 0.083 g/g, while theoretical estimates suggest a maximum yield of 0.32 g/g under conditions where all acetyl-CoA is converted to lipids, without biomass formation. Other reports indicate that lipid yields closer to 0.2 g/g are more commonly achieved (Papanikolaou and Aggelis 2011), suggesting that the efficiency of the bioprocess reached approximately 41.5%.

In a secondary screening, glucose was replaced with glycerol, a cost-effective alternative for media preparation (Lopes Da Silva et al. 2023). Robles-Iglesias et al. (2023) recommended glycerol as a suitable carbon source for lipid biosynthesis using yeast cell factories. Glycerol has been extensively studied both as the sole carbon source and in combination with glucose, with the C/N ratio identified as a key factor influencing the oleaginous character. High concentrations of glycerol (i.e., high C/N ratios) have been reported to suppress lipid degradation and prevent the assimilation of fatty acids under stress conditions (Dritsas and Aggelis 2023). When comparing three different C/N ratios, the highest total lipid concentrations were achieved at a C/N of 150:1. In contrast, a C/N ratio of 225:1 resulted in reduced lipid accumulation for *Y. lipolytica* LC015 and *Cl. lusitaniae* LC191 (Fig. 2), while the lowest lipid concentrations were observed at a C/N ratio of 75:1, consistent with previous findings Dobrowolski et al. (2016).

For *Y. lipolytica* LC015, high C/N ratios led to a non-oleaginous state, characterized by elevated biomass production (2.5 g/L for glucose, 5 g/L for glycerol). However, in a C/N ratio of 150:1 with glycerol, the oleaginous character was observed. Erian et al. (2022) reported five glycerol transporters in *Y. lipolytica*, suggesting that glycerol uptake occurs through passive diffusion at high concentrations. Furthermore, Da Cunha et al. (2019) reported that glycerol/$H^+$ symporters are activated under high glycerol concentrations, and repressed by glucose. The reduced accumulation of lipids observed under high C/N ratios suggests that lipogenic pathways may only be partially activated, or that fatty acids may be assimilated, contrasting with the hypothesis proposed by Dritsas and Aggelis (2023).

The lipid profile suggested core FAMEs that were present in all samples, independent of carbon source or strain. The presence of palmitic, stearic, oleic, and linoleic acids has been commonly reported as fatty acids in yeasts (Papanikolaou and Aggelis 2011; Mattanna et al. 2014; Tchakouteu et al. 2015; Poontawee et al. 2017; Carsanba et al. 2018; Mota et al. 2022). The composition of fatty acids is generally influenced by the yeast strain and the carbon source utilized during growth (Gientka et al. 2017). The results showed different lipid profiles depending on the carbon source. The suitability for each industry application depends on the ratio of Saturated and Unsaturated fatty acids. The results showed that most FAMEs (except *Cl. lusitaniae* LC126 and *Rhodotorula* sp. M6) are suitable for the biodiesel industries, according to the predictions of the models Ramírez-Verduzco et al. (2012) and Anahas and Muralitharan (2018) compared to the biodiesel standards.

Two uncommon fatty acids were identified: petroselinic acid (C18:1$\Delta$ $^6$), which is associated with specific clades of plants (Wang et al. 2024), and C16:1$\Delta$ $^{10}$, a rare fatty acid previously reported only in mutants of yeast desaturases (Gan et al. 2022). As indicated in Table 2, petroselinic acid was detected exclusively in *Aureobasidium* sp. grown on glycerol as a carbon source. Other species, such as *C. lusitaniae* or *P. rajashtanensis*, were also grown in glycerol but did not produce petroselinic acid, suggesting the presence of a specific $\Delta$ $^6$-ACP desaturase in *Aureobasidium* sp., along with active $\Delta$ $^9$ or $\Delta$ $^{12}$ desaturases, as previously reported (Gostinčar et al. 2008).

The presence of petroselinic acid is of significant importance, due to its melting point of 33 °C, which makes it solid but unsaturated at environmental temperatures, positioning it as a potential alternative to margarine production (Kazaz et al. 2022). Its cleavage into adipic (C6:0) and lauric (C12:0) acids suggests applications in the manufacturing of plastics and soaps. Furthermore, petroselinic acid can serve as a substrate for the production of sophorolipids Delbeke et al. (2016), as well as estolide esters and nutraceutical applications (Avato and Tava 2022). Moreover, this fatty acid has shown strong antibacterial and antifungal activities (Lee et al. 2022; Yoshino et al. 2022; Wang et al. 2024). As proposed by Wang et al. (2024), if petroselinic acid targets Fba1p (fructose-1,6-bisphosphate aldolase), its presence in *Aureobasidium* sp. under glycerol conditions may prevent self-antifungal activity by redirecting carbon flux from glycerol through the glycolysis pathway through dihydroxyacetone phosphate (Dobrowolski and Mironczuk 2020).

Methyl (Z)-heptadec-10-enoate (C16:1$\Delta$ $^{10}$) was detected in *Aureobasidium* sp. LC112 and *Cl. lusitaniae* LC126. Its presence probably involves an additional 16:0-ACP desaturase, as hypothesized for plants (Gan et al. 2022). The presence of these uncommon fatty acids in yeasts grown on glycerol as the sole carbon source suggests that specific desaturases are regulated by catabolite repression (Xue et al. 2017), as well as the down-regulation of the $\beta$ -oxidation pathway and increased flux through Glyceraldehide-3-Phosphate (G3P) production, which may be influenced by the availability of glycerol (Klein et al. 2017; Xue et al. 2017). Furthermore, to our knowledge, this study represents the first report of petroselinic and C16:1$\Delta$ $^{10}$ in yeasts and yeast-like fungi.

Regarding *Rhodotorula* sp. M6, the lipid profile revealed the presence of FAMEs containing 20 or more carbon atoms, consistent with previous reports in the literature. Among the detected long-chain fatty acids (LCFAs), arachidonic acid (C20:0) was the most abundant (Zhang et al. 2022; Wu et al. 2023), followed by cis-13,16-docosadienoic acid (DDA, C22:2 $\omega$6; Abaza et al. (2024)), Methyl 20-methyl-heneicosanoate (C23:0; Krikigianni et al. (2022); Wu et al. (2023)) and cis-8,4,11-docosatrienoic acid (DTA, C22:3 DTA $\omega$8; Shang et al. (2015)). Although 13-Docosenoic acid (a precursor of DDA) was also detected (see Table S1), it could not be quantified. DDA and DTA have been reported to exhibit strong anti-inflammatory, antitumor, and antioxidant properties (Chen et al. 2021), and considerable efforts have been made to improve their production in yeasts such as *Y. lipolytica* (Tang et al. 2024).

To our knowledge, this is the first report of DDA and DTA production in a wild strain of *Rhodotorula* sp. Considerable efforts have been made to increase LCFA production in various *Rhodotorula* strains through metabolic engineering of fatty acid biosynthetic pathways (Liu et al. 2021; Wu et al. 2023), starting with stearic acid as the primary precursor to FAME in this group. The ability of a naturally occurring strain to produce LCFAs suggests that bioprocess optimization could be used to enhance its production, potentially bypassing the need for genetically modified organisms (GMOs).

## Conclusion

This study successfully identified Colombian aquatic yeast strains capable of accumulating lipids, demonstrating their feasibility as microbial platforms for biotechnological applications. Among the 56 strains evaluated, 12 were considered oleaginous, with lipid yields exceeding 20% of dry biomass. Five strains -*Aureobasidium* sp. LC112, LC200, *Papiliotrema rajashtanensis* LE078, *Clavispora lusitaniae* LC126, and *Rhodotorula* sp. M6- stood out for their high lipid yield and biomass yields. Notably, *Aureobasidium* sp. LC112 demonstrated a lipid yield of 0.083 g/g glucose, achieving a high proportion of polyunsaturated fatty acids (PUFAs) with 75% of total lipids under glucose-based conditions. When cultured with glycerol as a carbon source, produced petroselinic acid and methyl (Z) -heptadec-10-enoate, it is positioned as a potential candidate for specialized oleochemical production, such as adipic or lauric acids, sophorolipids, or nutraceutical industries, such as margarines or functional foods. *Rhodotorula* sp. M6 exhibited long-chain fatty acids, emphasizing its potential for nutraceutical applications. These findings underscore the industrial relevance of aquatic yeasts as sustainable lipid producers with diverse fatty acid profiles suitable for biofuels, nutraceuticals, and high-value oleochemicals. Future research should focus on optimizing growth conditions, metabolic engineering, and scaling strategies to harness the full potential of these strains for commercial applications. Highlight efforts include (1) elucidating yeast adaptations to tropical aquatic ecosystems; (2) identifying desaturases responsible for the production of uncommon fatty acids; (3) optimizing culture conditions to enhance lipid yields; and (4) developing metabolic models to predict and maximize lipid production.

## Supplementary Information

Below is the link to the electronic supplementary material.

Supplementary file 1 (tif 135 KB)

Supplementary file 2 (tif 147 KB)

Supplementary file 3 (docx 28 KB)

## Data Availability

All data related to Figures 1 and 2, GC-MS raw reports and FAME quantification are available at DOI 10.17632/t34xzjy9ys.2
