# Supplementary material for: Tropical lakes as a novel source of oleaginous yeasts with lipid profiles for biodiesel, oleochemical, and nutraceutical applications
Source: World J Microbiol Biotechnol. 2025 Mar 13;41(3):105. doi: 10.1007/s11274-025-04309-7 (PMC11906551; doi:10.1007/s11274-025-04309-7)
Supplement: Supplementary file 3 — (docx 28 KB) [file 11274_2025_4309_MOESM3_ESM.docx]

Supplementary material

**Methods**

Chemical biodiesel quality parameters like cetane number (CN), iodine value (IV), saponification value (SV), and cold filter plugging point (CFPP) were calculated from Anahas et al. (2015), with the next equations (1 to 4):

$CN=46.3+\left( \frac{5458}{SV} \right)-(0.225\times IV)$ (1)

$SV=\Sigma\frac{(560\times N_{i})}{M_{i}}$ (2)

$IV= \Sigma\frac{(254\times{D_{i}\times N}_{i})}{M_{i}}$ (3)

where *D* is the number of double bonds, *M* is the FA molecular mass, and *N* is the percentage of each FA component (i).

$CFPP=\left( 3.1417\times LCSF \right)-16.477$ (4)

Other physical parameters like viscosity (*υ*) (equation 5), and density (*ρ*) (equation 6), also critical for the fuel quality of the biodiesel were predicted from the FAME profiles according to Ramirez-Verduzco et al. (2012).

$\ln\left( \eta_{i} \right)=-12.503+2.496\times\ln\left( M_{i} \right)-0.178\times D_{i}$ (5)

where, $\eta_{i}$ is the kinematic viscosity of at 40 °C in mm^2^ s^−1^; *ρ*_i_ is the density at 20 °C in g/cm^3^;

$\rho_{i}=0.8463+\frac{4.9}{M_{i}}+0.0118\times D_{i}$ (6)

where *ρ_i_* is the density at 20 °C of the *i*th FAME in g/cm^3^.

To assess the total lipid profile for each strain, the equation (7) was used:

$f_{y}=\sum_{i=1}^{n} z_{i}\times f_{i}$ (7)

where *f* is a function that represents any physical property (the subscripts *b* and *i* refer to the yeast and the pure *i*th FAME, respectively), *z_i_* is the percentage area of the *i*th FAME. The function *f_y_* must be replaced by each predicted variable η*_y_* or *ρ_y_*.

**Table S1.** Fatty Acids Methyl Esters (FAMEs) area percentage (wt%) and predicted chemical parameters for biodiesel suitability.

| **Strain** | **Carbon source** | **C/N ratio** | **FAMES (corrected % Area)** | | | | | | | | | | | | |  |  |  |  |  |  |  |  |
| --- | --- | --- | --- | --- | --- | --- | --- | --- | --- | --- | --- | --- | --- | --- | --- | --- | --- | --- | --- | --- | --- | --- | --- |
|  |  |  | **S** | **S** | **MU** | **MU** | **S** | **MU** | **MU** | **PU** | **S** | MU | **PU** | **PU** | **S** | **CN** | **SV** | **IV** | **DU** | **LCSF** | **CFPP** | **υi** | **ρ** |
|  |  |  | **C14:0** | **C16:0** | **C16:1Δ9** | **C16:1Δ10** | **C18:0** | **C18:1Δ6** | **C18:1Δ9** | **C18:2Δ9,12** | **C20:0** | **C22:1Δ13*** | **C22:3Δ8,11,14** | **C22:2Δ13,16** | **C23:0** |  |  |  |  |  |  |  |  |
| **M (Molecular mass g/mol)** |  |  | **242,975** | **270,4507** | **268,4348** | **268,4348** | **298,5038** | **296,4879** | **296,4879** | **294,4721** | **326,557** | **352,5943** | **320,51** | **350,58** | **354,6** |  |  |  |  |  |  |  |  |
| **D (number of insaturations)** |  |  | **0** | **0** | **1** | **1** | **0** | **1** | **1** | **2** | **0** | **1** | **3** | **2** | **0** |  |  |  |  |  |  |  |  |
| LC112 | Glucose | 300:1 | 7,96 | 20,74 | 1,49 |  | 5,48 |  | 42,74 | 21,59 |  |  |  |  |  | 57,14 | 196,46 | 75,27 | 87,42 | 4,81 | -1,36 | 4,32 | 0,87 |
| LC112 | Glycerol | 225:1 |  | 18,64 | 4,50 | 0,67 | 6,43 | 1,02 | 44,18 | 24,56 |  |  |  |  |  | 57,03 | 181,46 | 85,99 | 99,50 | 5,08 | -0,52 | 4,37 | 0,87 |
| LC112 | Glycerol | 150:1 |  | 16,06 | 5,18 | 0,45 | 4,80 | 1,71 | 45,51 | 26,29 |  |  |  |  |  | 55,43 | 184,19 | 91,13 | 105,43 | 4,00 | -3,90 | 4,34 | 0,88 |
| LC200 | Glucose | 300:1 |  | 20,54 | 1,59 |  | 3,68 |  | 39,22 | 34,97 |  |  |  |  |  | 53,06 | 193,33 | 95,43 | 110,75 | 3,89 | -4,25 | 4,29 | 0,88 |
| LC200 | Glucose | 300:1 |  | 19,64 | 1,62 |  | 3,34 |  | 37,45 | 37,96 |  |  |  |  |  | 52,25 | 193,22 | 99,09 | 114,98 | 3,63 | -5,06 | 4,26 | 0,88 |
| LC200 | Glucose | 300:1 |  | 19,94 | 1,31 |  | 3,60 |  | 37,96 | 37,19 |  |  |  |  |  | 52,52 | 193,20 | 97,92 | 113,65 | 3,79 | -4,56 | 4,27 | 0,88 |
| LC126 | Glucose | 300:1 |  | 29,91 |  |  | 53,24 |  | 9,76 | 7,09 |  |  |  |  |  | 69,84 | 193,73 | 20,60 | 23,94 | 29,61 | 76,55 | 5,00 | 0,87 |
| LC126 | Glycerol | 225:1 | 0,29 | 18,03 | 4,39 | 0,71 | 6,17 |  | 45,37 | 25,04 |  |  |  |  |  | 54,95 | 193,53 | 86,89 | 100,55 | 4,89 | -1,12 | 4,36 | 0,88 |
| LE078 | Glycerol | 75:1 |  | 27,49 |  |  | 5,48 |  | 12,61 | 54,43 |  |  |  |  |  | 50,80 | 194,51 | 104,70 | 121,47 | 5,49 | 0,76 | 4,15 | 0,88 |
| M6 | Glucose | 300:1 |  | 30,21 |  |  | 24,06 |  | 21,91 | 13,25 | 0,73 | 4,56 | 3,84 | 0,84 | 0,60 | 62,33 | 191,77 | 55,25 | 62,32 | 16,88 | 36,55 | 4,80 | 0,87 |
| M6 | Glucose | 300:1 |  | 28,50 |  |  | 18,78 |  | 26,79 | 13,28 | 0,78 | 5,55 | 4,28 | 1,24 | 0,79 | 60,97 | 190,98 | 61,84 | 69,95 | 14,19 | 28,11 | 4,79 | 0,87 |
| ρ |  |  | 0,866 | 0,864 | 0,876 | 0,876 | 0,863 | 0,875 | 0,875 | 0,887 | 0,861 | 0,872 | 0,897 | 0,884 | 0,860 |  |  |  |  |  |  |  |  |
| ln(υ*i)* |  |  | 1,207 | 1,475 | 1,278 | 1,278 | 1,721 | 1,526 | 1,526 | 1,331 | 1,945 | 1,959 | 1,365 | 1,767 | 2,151 |  |  |  |  |  |  |  |  |
| υi |  |  | 3,345 | 4,370 | 3,590 | 3,590 | 5,591 | 4,601 | 4,601 | 3,786 | 6,996 | 7,091 | 3,915 | 5,851 | 8,593 |  |  |  |  |  |  |  |  |

**Table S2.** Fatty Acids Methyl Esters (FAMEs) general characterization (corrected percentage area) for oleaginous yeasts grown in glucose or glycerol as carbon source. SFA = Saturated Fatty Acid; MUFA = Monounsaturated Fatty Acid; PUFA = Polyunsaturated Fatty Acid; UFA = Unsaturated Fatty Acid.

| **Strain** | **Carbon source** | **C/N ratio** | **SFA** | **MUFA** | **PUFA** | **UFA** |  |
| --- | --- | --- | --- | --- | --- | --- | --- |
|  |  |  |  |  |  |  |  |
|  |  |  |  |  |  |  |  |
| LC112 | Glucose | 300:1 | 34,2 | 44,2 | 21,6 | 65,8 |  |
| LC112 | Glycerol | 225:1 | 25,1 | 50,4 | 24,6 | 74,9 |  |
| LC112 | Glycerol | 150:1 | 20,9 | 52,9 | 26,3 | 79,1 |  |
| LC200 | Glucose | 300:1 | 24,2 | 40,8 | 35,0 | 75,8 |  |
| LC200 | Glucose | 300:1 | 23,0 | 39,1 | 38,0 | 77,0 |  |
| LC200 | Glucose | 300:1 | 23,5 | 39,3 | 37,2 | 76,5 |  |
| LC126 | Glucose | 300:1 | 83,1 | 9,8 | 7,1 | 16,9 |  |
| LC126 | Glycerol | 225:1 | 24,5 | 50,5 | 25,0 | 75,5 |  |
| LE078 | Glycerol | 75:1 | 33,0 | 12,6 | 54,4 | 67,0 |  |
| M6 | Glucose | 300:1 | 55,0 | 26,5 | 17,9 | 44,4 |  |
| M6 | Glucose | 300:1 | 48,1 | 32,3 | 18,8 | 51,1 |  |
| TOTAL | | | 35,9 | 36,2 | 27,8 | 64,0 |  |
